# Remote Food Security Research Project: Applying an Indigenist Research Lens

**DOI:** 10.1002/hpja.954

**Published:** 2025-01-23

**Authors:** Ellie Chan, Caroline Deen, Kani Thompson, Emma Stubbs, Amanda Webb, Abdolvahab Baghbanian, Yvonne Cadet‐James

**Affiliations:** ^1^ Public Health Central Australian Aboriginal Congress Alice Springs Northwest Territories Australia; ^2^ School of Public Health the University of Queensland Herston Queensland Australia; ^3^ Population Health Apunipima Cape York Health Council Cairns Queensland Australia; ^4^ Poche Centre for Indigenous Health The University of Sydney Sydney New South Wales Australia; ^5^ University Centre for Rural Health The University of Sydney Sydney New South Wales Australia; ^6^ Indigenous Education and Research Centre James Cook University Cairns Queensland Australia

**Keywords:** aboriginal, food security, indigenist research, indigenous, indigenous methodologies, Torres Strait islander

## Abstract

This paper aimed to reflect on how Rigney's model of Indigenist research informed the research design of a project which explored community‐led solutions to improve food security in remote Aboriginal and Torres Strait Islander communities. The project was conducted in partnership with two Aboriginal Community Controlled Health Organisations (ACCHOs); Apunipima Cape York Health Council (Apunipima) and Central Australian Aboriginal Congress (Congress), communities in Central Australia and Cape York, Queensland and researchers from the University of Queensland, Monash University, Dalhousie University and Menzies School of Health Research. On reflection the principles of Indigenist research were evident providing a means of resistance to oppression through Indigenous stakeholders being in control of research to address social determinants, in this case food security. Aboriginal and Torres Strait Islander world views, lived experiences and knowledges were embedded in the research and informed governance, implementation and knowledge translation. ACCHOs and communities gained a political voice through advocacy and actions at the local, state and national levels. The development of a Community Framework led by ACCHOs and community stakeholders to address food security serves to talk to the three principles of ‘Resistance, Political Integrity and Privileging Indigenous Voices’.

## 1 Remote Food Security Project

This multi‐phase study aimed to explore community‐led solutions for improving food security in remote communities in Central Australia and Cape York. Phase 1 evaluated the impact of price discounting healthy food and drinks on diet quality in women and children, and affordability of a healthy diet. In‐depth interviews with community members captured their lived experiences including factors influencing food insecurity, coping strategies and potential solutions.

Phase 2 aimed to develop a community framework for improving household and community food security. Photovoice methodology was implemented in communities, giving voice to people's lived experiences and proposed solutions for improving food security. Community prioritisation workshops were held in these communities, where stakeholders came together to hear project findings, and to identify priorities and potential solutions with the greatest impact. A knowledge exchange forum then bought representatives from 10 communities in Cape York and Central Australia together to consider collective priorities and solutions, to inform a community‐led framework and a policy translation plan. The methodology and outcomes of Phase 1 and Phase 2 are described elsewhere [1].

## 2 Indigenist Research Framework

While a previous publication [1] reported on the co‐design process which considered Indigenous peoples' ways of ‘Knowing, Being and Doing’ [2] using the CREATE Tool [3] and informed by the Research for Impact Tool [4], the intent of this paper was to consider the research design from an Indigenist research methodology lens. This group refection included Aboriginal and Torres Strait Islander and non‐Indigenous members of the research and ACCHO project teams.

The research partners wanted to ensure that the approach to research design was embedded in understanding and respecting the historical perspective; the political positioning of Indigenous peoples; human rights and social justice; diverse epistemologies, ontologies and axiologies and knowledge systems. The work of Aboriginal scholar, Lester‐Irabinna Rigney who introduced Indigenist research informed the research design, emphasising, ‘…three fundamental and interrelated principles:

- Resistance as the emancipatory imperative in Indigenist research
- Political integrity in Indigenous research
- Privileging Indigenous voices in Indigenist research’ [5, p. 116].

These principles were central to the project, recognising the struggle of Aboriginal and Torres Strait Islander peoples from the ongoing impact of colonisation, but also acknowledging the strength, survival and resistance of people to take control of their destiny. In terms of political integrity, Rigney [5, p. 117] states, ‘Indigenous Australians have to set their own political agenda for liberation. To the extent that research contributes to that agenda, it must be undertaken by Indigenous Australians. There must be a social link between research and the political struggle of our communities’. In this project there is a direct social link between food insecurity and its impact and the political control and oppression by past and current Government legislation and policies.

Rigney also states that research must be undertaken by those who live and understand the struggle. ‘Indigenist research focuses on the lived, historical experiences, ideas, traditions, dreams, interests, aspirations, and struggles of Indigenous Australians. Indigenist research gives voice to Indigenous people’ [5, p. 118]. This project was born out of concern, advocacy and previous work on food insecurity by Congress and Apunipima on behalf of communities in Central Australia and Cape York. So even prior to the first research concept planning meeting, Indigenous voices were at the forefront of this research.

## 3 Leadership and Governance

Indigenous voices continued through governance and leadership at each stage of the project, as chief and associate investigators, representation on the governance committee, project teams, Community Advisory Groups (CAGs) and implementation and translation teams. CAGs were established in each participating community to provide guidance and support to the research team on engagement, cultural and community governance, protocols and feedback processes. This was complimented by community researchers employed in each community. The CAGs and community researchers' strong cultural identities, traditional and local knowledges was a crucial factor in ensuring cultural determinants informed the research [6, p. 181]. The CAGs lived experiences were instrumental in identifying issues with proposed research methods due to what was happening in communities at the time and worked with researchers and communities to find appropriate solutions.

## 4 Cultural Safety and Responsiveness

‘Cultural safety is the positive recognition and celebration of cultures. It is more than just the absence of racism or discrimination and more than “cultural awareness” and “cultural sensitivity.” It empowers people and enables them to contribute and feel safe to be themselves’ [7]. From a research ethical perspective, Congress [8], and Apunipima [9] through community consultation had previously implemented research principles relevant to the context of their organisations and communities. These principles were based on the National Health and Medical Research Council (NHMRC) core values of ‘spirit and integrity, cultural continuity, equity, respect and responsibility’ [10]. The employment of local community researchers was paramount for cultural safety at the ground level. Acknowledgement of differences between communities and across regions was essential, and the need to be flexible and adaptable key to operating within a culturally safe framework. Early consideration of these factors resulted in a budget which catered for the complexity of working in remote communities. This allowed for changes to project methods and timelines in response to community needs and priorities.

## 5 The Cultural Interface

The project partners are from diverse social, cultural and ethnic backgrounds working together in the cultural interface. Nakata describes this space as follows [11, p. 9]:

In this space are histories, politics, economics, multiple and interconnected discourses, social practices and knowledge technologies which condition how we come to look at the world, how we come to know and understand our changing realities in the everyday, and how and what knowledge we operationalise in our daily lives. Much of what we bring to this is tacit and unspoken knowledge, those assumptions by which we make sense and meaning in our everyday world.

Respecting this diversity and potential for misunderstanding and conflict was acknowledged in initial planning informing the governance model and the methodology. Several situations occurred where the political freedom for everyone to speak openly resulted in researchers, ACCHO staff and community coming together to better understand the often complex community context informed by history, culture and relational ways of working which resulted in agreed changes to the way certain aspects of the research were applied.

## 6 Partnerships and Relationships

Partnerships were identified by stakeholders as a strength, including relationships between the research collaborative and the research team and the communities. The partnership between Congress and Apunipima, provided a valuable opportunity to learn from one another and work collectively toward addressing a shared priority. Factors which supported positive relationships included transparent communication, strong leadership within each organisation, accountability across the project, and equal valuing of knowledge from all stakeholders. ACCHOs with longstanding relationships with communities leading the implementation of the research promoted progressive strengthening of relationships. Project staff's frequent presence in communities supported the maintenance of relationships between CAGs, community researchers and members.

## 7 Knowledge Translation

Stakeholders highlighted that the robust sharing and learning facilitated through partnerships and relationships stood out as a distinctive aspect of their research experience. This observation emerged during a two‐day Knowledge Exchange Forum integral to the research process. Decisions about data sovereignty came from stakeholders regarding what data was to be collected, how it was used and ownership of the data, informed initially by ACCHO research principles then validated by CAGs. Integrated knowledge translation occurred throughout the process of disseminating community and regional level findings back to communities, the ACCHOs including Boards, Health Action Teams and other stakeholders to inform future stages of the project. CAGs directed the most appropriate way to present community findings throughout and on conclusion of the project, to inform local action and advocacy. Some community representatives have already used the results to begin discussions about developing a community owned garden business and the possibility of local securing, butchering and selling of meat. One community member has engaged with the media to share their food security story and others have reported that they have used their community results to present at a local ‘Cost of Food Summit’. These types of initiatives address food insecurity directly and indirectly, impacting on the broader elements such as employment, economic development, empowerment, and community ownership.

The project remains committed to translating findings into policy and practice by leveraging available state and national avenues, guided by community. This included incorporating project findings into a submission for the National Inquiry into Food Security in Australia and insights gained from the Knowledge Exchange Forum were used to shape a response to the draft Queensland Remote Food Security Strategy. The project team is also working in partnership with community representatives, Aboriginal Medical Service Alliance Northern Territory, Queensland Aboriginal and Islander Health Council and National Indigenous Australians' Agency to ensure that community representatives have their voice heard in the development of a national strategy for food security in remote communities.

## 8 Empowerment, Knowledge Sharing and Learning

A focus of the research was two way knowledge sharing and learning between all stakeholders. Acknowledging stakeholder strengths assisted in identifying areas to strengthen capacity and capability and build on empowerment to achieve outcomes. ACCHOs were able to apply their research capabilities in the food security space, benefiting from engagement in different research designs, methodologies and processes. Researchers benefited from working in a two way peer learning environment in the cultural interface being mentored by CAGs, community researchers and members. ACCHO project staff were able to strengthen research skills through leading the implementation of the study, and contributing to publications, conference presentations, and other activities. Employment of local community researchers provided employment and opportunity for community members to further develop research skills including data collection and analysis and learn more about food security.

## 9 Conclusion

This paper aimed to reflect on how Indigenist research principles informed the design and implementation of a Remote Food Security Project based on Rigney's three principles, ‘Resistance as the emancipatory imperative, Political integrity and Privileging Indigenous voices’. Understanding of the historical context highlighted the ongoing impact of colonisation resulting in oppression and the social determinants of health with food security as an example. In terms of resistance as the emancipatory imperative, this research acknowledged the survival of Aboriginal and Torres Strait Islander people and ensured their strengths, lived experiences and knowledges underpinned all aspects of the research as determined by them. The research provided a means for Aboriginal and Torres Strait Islander people to set their own political agenda by having a voice in advocating for improved food security in remote Indigenous communities. Data sovereignty meant that stakeholders had control over what data was collected, its use and ownership of the data, which fed into knowledge translation plans and activities at local, state and national level. Indigenous governance and leadership at all stages of the research was at the centre of the research methodology not only giving voice to those involved but control over the research process and their own research agendas, important in the fight for self‐determination.

## Conflicts of Interest

The authors declare no conflicts of interest.

## Data Availability

Data sharing is not applicable to this article as no new data were created or analyzed in this study.
